# The Potential Diagnostic and Prognostic Value of Circulating MicroRNAs in the Assessment of Patients With Prostate Cancer: Rational and Progress

**DOI:** 10.3389/fonc.2021.716831

**Published:** 2022-02-04

**Authors:** Elham Samami, Ghazaleh Pourali, Mahla Arabpour, Azar Fanipakdel, Soodabeh Shahidsales, Seyed Alireza Javadinia, Seyed Mahdi Hassanian, Saeid Mohammadparast, Amir Avan

**Affiliations:** ^1^ Network of Immunity in Infection, Malignancy and Autoimmunity (NIIMA), Universal Scientific Education and Research Network (USERN), Tehran University of Medical Sciences, Tehran, Iran; ^2^ Cancer Research Center, Mashhad University of Medical Sciences, Mashhad, Iran; ^3^ Metabolic Syndrome Research Center, Mashhad University of Medical Sciences, Mashhad, Iran; ^4^ Vasei Clinical Research Development Unit, Sabzevar University of Medical Sciences, Sabzevar, Iran; ^5^ Department of Cell, Developmental and Integrative Biology, University of Alabama at Birmingham, Birmingham, AL, United States; ^6^ Basic Medical Sciences Institute, Mashhad University of Medical Sciences, Mashhad, Iran

**Keywords:** Prostate cancer, miRNA, Circulating biomarker, Prognostic factor, Therapeutic targets

## Abstract

Prostate cancer (P.C.) is one of the most frequent diagnosed cancers among men and the first leading cause of death with an annual incidence of 1.4 million worldwide. Prostate-specific antigen is being used for screening/diagnosis of prostate disease, although it is associated with several limitations. Thus, identification of novel biomarkers is warranted for diagnosis of patients at earlier stages. MicroRNAs (miRNAs) are recently being emerged as potential biomarkers. It has been shown that these small molecules can be circulated in body fluids and prognosticate the risk of developing P.C. Several miRNAs, including MiR-20a, MiR-21, miR-375, miR-378, and miR-141, have been proposed to be expressed in prostate cancer. This review summarizes the current knowledge about possible molecular mechanisms and potential application of tissue specific and circulating microRNAs as diagnosis, prognosis, and therapeutic targets in prostate cancer.

## Introduction

Prostate cancer (P.C.) served as the second most frequent with 37.5 per 100,000 incidence in countries with a higher development index and the fifth most leading to death cancer of men with 1.4 million new cases and 375,000 deaths, worldwide in 2020 (1). The main goal in the diagnosis of P.C. is to detect cancer early and treat patients at initial stages to raise cure rates. Prostate-specific antigen (PSA) testing, a highly sensitive diagnostic test for detection of P.C., influences the diagnosis of this medical condition by early detection of prostate cancer; however, its use declined recently (1). The lack of specificity and sensitivity in the PSA index provides the demand for rectified biomarkers. In the same way, personalized medicine using genes and proteins, i.e., specific features of cells, attracts attention of clinicians and researchers in the management of cancers including P.C. (2). It is becoming manifested that microRNAs (miRNA) are associated with progression and development of prostate cancer (3–8). MiRNAs are small regulatory RNAs with an average length of 22 nucleotides that affect the expression of their target genes and their dysregulation. They play a critical role in various biological functions, maintaining hemostasis, and due to their modulatory operations, they regulate near 60% of all human genes (5).

Moreover, miRNAs are found commonly in cancer-related genomic loci or fragile sites; thus, they implicate in carcinogenesis as a result of their involvement in the expression or suppression of many oncogene genes (9). Thus, they can be used as non-invasive specific markers for estimating the prognosis of disease and classification of tumor (10, 11). MiRNAs are detectable in different body parts, including body fluids (plasma/serum/urine) and tissues. Endogenous RNase cleaves miRNAs; however, having this capability to affect single or several gene targets simultaneously results in the regulation of numerous targets in the malignant cells.

There are a lot of mechanisms that cause cancer progression through modulation of miRNAs, such as deletions, amplifications, and mutations involving miRNA loci, epigenetic silencing, the dysregulation of transcription factors that affects specific miRNAs, or the inhibition of processing. Thus, the miRNA expression profile plays a critical role as a diagnostic and prognostic tool in malignant cancers (12). Plasma and serum samples of normal participants and non-recurrent and recurrent metastatic prostate cancer were assessed for 742 microRNAs by Bryant et al. using a real-time polymerase chain reaction analysis (RT-PCR) to identify differentially quantified microRNAs confirmed considerable changes in the concentration of twelve microRNAs in these patients comparing normal ones (13).

This review summarizes the current knowledge about possible molecular mechanisms and potential application of tissue-specific and circulating microRNAs as diagnosis, prognosis, and therapeutic targets in prostate cancer.

## Data Sources and Search Strategies

Searching for articles without a time limit was performed on international databases such as Science Direct, PubMed, and Google Scholar. To maximize the search consistency, we used the search terms “prostate cancer,” “miRNA,” and “circulating marker” with all possible combinations of OR and AND operators.

## Study Selection

Initially, the authors provided a list of all the articles’ titles and abstracts in the above databases. After an initial review of articles’ abstracts, related topics were selected, and non-related studies were excluded. Finally, the list of references used in all the searched articles was reviewed to include other possible sources. Those matched with inclusion criteria were selected. The main inclusion criteria in this study were assessing the role of miRNAs in cancer, which were utilized in the introduction section, and the papers with a specific focus on prostate cancer for the rest of this article. Exclusion criteria included case reports, editorials, letters to editor, and vague and unclear studies or the subject of the study was not relevant.

## Computational Methods for Predicting Non-Coding RNAs

The miRNAs are essential non-coding RNAs and play significant roles in lots of biological processes; thus, many methods have been developed for predicting disease-related miRNAs and their functions, such as informatic and experimental methods. However, experimental methods are not only long-delayed but also expensive. Genomic SELEX, microarray analysis, and parallel cloning of ncRNAs by specific cDNA libraries or enzymatic and chemical RNA sequencing belong to this category (14). Therefore, hundreds of computational methods were developed. Computational methods have been proposed to analyze the non-coding RNAs. We introduced, discussed, and analyzed these methods in this section. Homology-based methods (including sequence-based method, structure-based methods, and hybrid methods), *de novo* methods using RNA sequence and structure features (including sequence feature-based methods, structure feature-based methods, and hybrid feature-based methods), transcriptional sequencing and assembling-based methods, and RNA family-specific methods (including miRNA-specific methods and lncRNA) are among the four most available methods of computational methods in analyzing non-coding RNAs (15). Recently, some computational methods have been proposed, such as iMiRNA-PseDPC (16), iMiRNA-SSF (17), miRNA-dis (18), miRNA-deKmer (14), and 2L-piRNA (19).

In addition, all the latest biological knowledge paved the way for developing the new bioinformatics tools, algorithms, and miRNA-specific databases designed for non-coding miRNA prediction (20). Recently, it has been an emerging interest in predicting individual outcomes as a result of genomic alterations (21). Machine learning is defined as the subfield of AI (artificial intelligence).

These methods can accelerate the miRNA studies. Similarly, Lee et al. designed a prediction model by means of ML and bioinformatics tools and based on the genomic study that predict late toxicity after radiotherapy (22).

## Circulating MiRNA as Prognostic and Diagnostic Biomarkers

**Figure 1:**
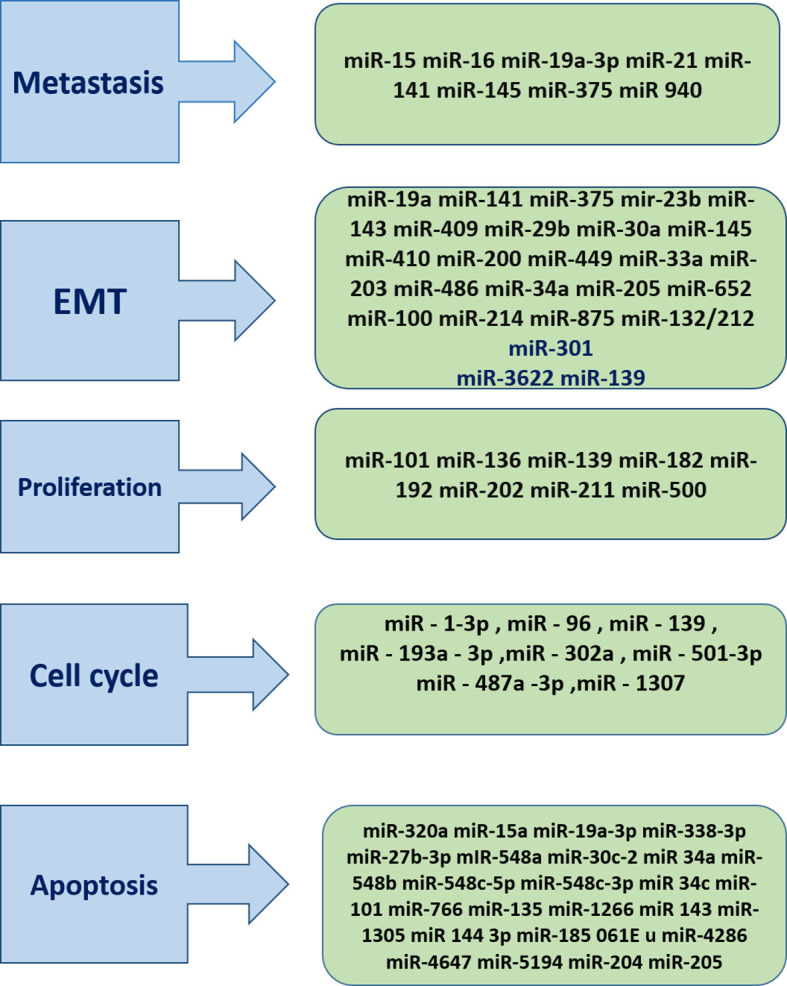
miRNAs Play critical role in cancer progression .As it has been described miRNAs are presented in different stage of cancer. Hence they can be target of the therapeutics and diagnostics approaches.

Alternations in miRNA regulation, i.e., upregulation or downregulation, during carcinogenesis, tumor progression, and metastatic transformation, have been reported in several investigations of tumors, including prostate (23–34). The association between miRNA activity and the steps of prostate cancer has been demonstrated (**Figure 1**) (5). Sylvestre et al. assessed the expression of miR-20a in the P.C. cell line and its correlation with apoptosis, showing that miRNA has a potential antiapoptotic role. Any inhibition of miR-20a resulted in increased cell death. However, its overexpression in doxorubicin-treated P.C. led to a 2-fold decrease in cell death and increased survival by 2 in that cell line (35). In a study by Agaoglu et al., plasma levels of miRNAs including miR-21, miR-141, and miR-221 were measured by real-time PCR in the plasma of fifty one pathologically confirmed P.C. patients in two subgroups, with localized/local advanced or metastatic P.C. and twenty healthy people (23). Agaoglu et al. report that amounts of two of these cancer-related miRNAs, i.e., miR-21 and miR-221, were significantly higher in patients than in normal people. These miRNAs had increased dramatically in metastatic patients than in patients with localized/local advanced disease; however, the results were most considerable for miR-141. The study finally concluded that among these miRNAs, miR-21, and miR-141 are the two most valuable measurements, the former functions better as a diagnostic tool, i.e., for discrimination of P.C. patients from healthy controls, and the latter serves better as a prognostic tool for determination of the stage of disease (23).

The assessment of circulating microRNAs in the autochthonous mouse models and patients with metastatic castration-resistant prostate cancer (mCRPC) by Selth et al. showed an alternation of mmu-miR-141, mmu-miR-298 and mmu-miR-375, mmu-miR-346 levels in both groups. The upregulation of the first three of these microRNAs in the cancerous tissue has been evident in P.C. compared with normal prostate tissue. It seems that by progression of disease, mmu-miR-141, mmu-miR-298, and mmu-miR-375 are released into the serum. Furthermore, there was a positive correlation between presence of hsa-miR-141 and hsa-miR-375 in the cancerous tissue and the possibility of biochemical relapse of P.C. suggests the role of hsa-miR-141 and hsa-miR-375 in prostate cancer pathophysiology (26).

Serum samples of about 600 participants including healthy people and patients with different types of cancers with different stages were evaluated by Lodes et al. in terms of miRNA expression patterns (24). All cancer samples were from patients with carcinoma of breast, male and female genitourinary system (prostate and ovary), gastrointestinal organ (colon), and the lung. Based on the results of Lodes et al., overexpression of different miRNAs was reported in the serum of patients with advanced-stage P.C. in comparison to serum of normal participants. They point out these upregulated miRNAs as “miR-16, -92a, -103, -107, -197, -34b, -328, -485-3p, -486-5p, -92b, -574-3p, -636, -640, -766, -885-5p”. Also, a modest elevation in the signal for miR-141 was recorded in patients suffering from stage III–IV PC consistent with a previous report by Mitchell et al. (24, 25). Plasma miR-141 represents various functions in different studies. Mitchell et al. showed its value as a diagnostic tool for discrimination of P.C. patients; however, Agaoglu et al. showed its role as a prognostic measurement as it can distinguish metastatic prostate cancer patients from other stages (23–25). In a study by Fredsøe et al., they assessed the expression of 92 selected miRNAs in plasma samples of 753 patients with different stages of P.C. and non-cancer controls. They found various dysregulations of miRNAs with the overlap of 59 dysregulated miRNAs between BPH versus advanced P.C. and localized P.C. versus advanced P.C. They also identified four miRNA diagnostic ratio models, named *bCaP* (miR-375*miR-33a-5p/miR-16-5p*miR-409-3p) (36).

Rajendiran et al. found that miR-940 serum levels were significantly higher in cancer patients, especially those with clinically advanced tumors. This study also showed that miR-940 in combination with PSA has a higher value than miR-940 alone for the diagnosis of prostate cancer (37).

## Circulating MiRNAs as Predictive Biomarkers in Response to Therapy

Response assessment during treatment is essential to discriminate against the patient who benefits from treatment. Many efforts have been made; however, there is no agreement on the approach of choice. TaqMan Human MicroRNA Arrays and RT-PCR were used by Nguyen et al. to assess serum miRNAs of patients with different groups of P.C. including low-risk localized and high-risk localized disease and mCRPC to find the roles of these negatively regulated genes during carcinogenesis and tumor progression. They reported that P.C. has unique miRNA signatures even in its different subgroups. With disease progression from low-risk disease to mCRPC, increased serum levels of miR-375, -378, and 141 were observed (38). Considering previous reports supporting the results of Nguyen et al., they strongly suggest that the levels of miR-375 and miR-141 in the blood serum are two potential markers for follow-up and surveillance of patients with P.C. (38, 39). Newly diagnosed patients with metastatic hormone-sensitive prostate cancer were enrolled by Cheng et al. for measurement of the circulating miR-141, -200a-b, -210, and -375 by RT-PCR before and after (at 13 weeks) treatment (40). They showed the prognostic and predictive values of a new miRNA, miR-375, in the assessment of treatment of P.C. and confirmed the prognostic role of previously known miRNAs, miR-141 and miR-200a, in this disease. In another effort, Corcoran et al. assessed the value of miR-34a in the prediction of response to chemotherapy in patients with P.C. In their study, they enrolled P.C. patients with and without resistance to docetaxel and found that detection of miR-34a in exosomes is strongly correlated with response to the treatment (41). A study by Lin et al. found that the measurement of circulation levels of miR-200b and miR-20a is beneficial for predicting response to docetaxel in patients with P.C. (42). In a study by Benoist et al., miR-3687 was identified as a prognostic marker for response to enzalutamide in patients with mCRPC, and they confirmed the prognostic importance of miR-375 (43). The expression of five miRNAs (miR-93-5p, -125b-1-5p, -141-3p, -221-3p, and miR-375-3p) was assessed in 84 mCRPC patients in two groups treated with docetaxel and abiraterone by Zedan et al. They found that plasma levels of miR-141-3p and miR-375-3p can predict time to progression in mCRPC patients treated with docetaxel or abiraterone (44). Their results were a primary proof showing the potential benefit of circulating miRNAs as predictive biomarkers in response to therapy.

## MiRNAs as Therapeutic Targets

Granulin (GRN) has a critical role in different cancers, and its products cause malignant transformation, induce metastases, and interfere with antiapoptotic processes (45, 46). Recent studies have shown that as GRN is upregulated, miR-107 gets downregulated, and there is an inverse correlation between them. A study by Wang et al. showed that miRNAs including miR-15/107 can potentially target GRN mRNA; i.e., by transfection of GRN protein into P.C. cells, different members of the miR-107 gene group suppress the level of this protein. Hypothetically, it is possible that the expression of GRN can be attenuated by positive manipulation of miR-15/107 gene group expression. It potentially reduces the malignant transformation and metastases and subsequently regulates the antiapoptotic processes of GRN productions (28). The oncogenic effects of overexpression of MiR-21 have been indicted in many human cancers. The effects of MiR-21 on the signal transducer and activator of transcription 3 (STAT3) pathway have been reported by Yang et al., revealing that “miR-21 can be upregulated by IFN and that manipulation of miR-21 expression by miR-21 knockdown can be employed to enhance IFN’s apoptotic action” (29).

## Conclusions

**Figure 2:**
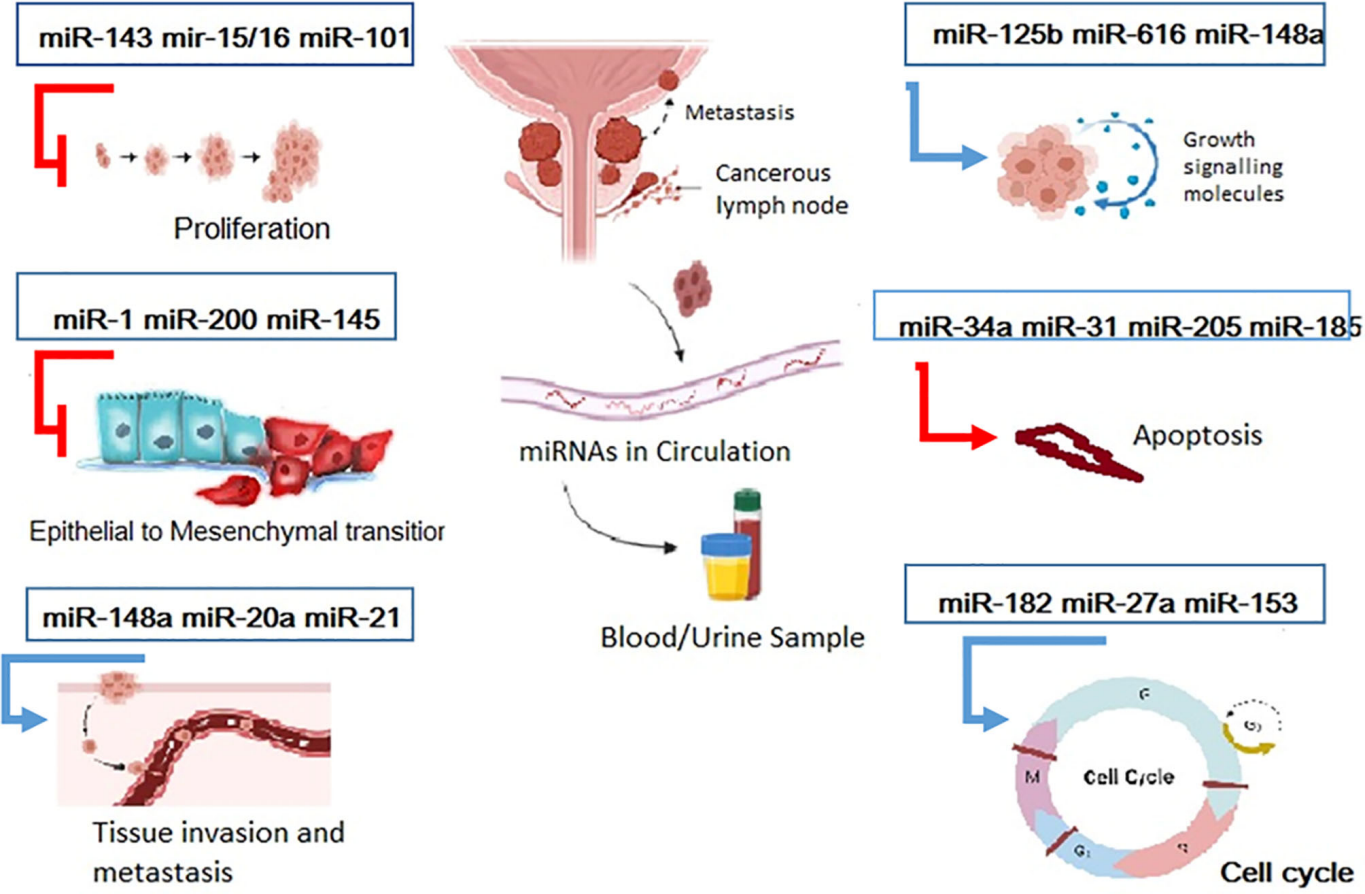
Overview of several mirNAs involved in both two different pathways for tumor progression and tumor suppression. Some miRNAs Promote cancer progression through induction of growth signals, tissue invasion and manipulation of cell cycle ,hence they called onco-miRNAs. On the contrary some miRNAs play protective role against cancer by inhibition of proliferation, EMT initiation of apoptosis. So this tumor suppressor mirs can be target as a therapeutic approaches.

Prostate cancer is one of the most frequently diagnosed cancers among men and the first leading cause of death worldwide. PSA is the only available standard method to assess patients’ response to treatment and follow-up. However, it has a lot of limitations, although there are several diagnostic and prognostic markers without consensus regarding them. Recently, dysregulations of miRNAs have been introduced as potential biomarkers for the diagnosis and assessment of prognosis of patients with P.C. and prediction of their response to treatment which can be found as chromosomal level or in the circulation. There are a lot of pitfalls in our knowledge about the molecular basis of P.C., and future investigations should be conducted to find out the role of dysregulations of miRNAs during carcinogenesis and progression of tumors to their advanced stages. The recent trends have also shown that the non-coding RNA analysis should also focus on the sequence composition, and several tools have been proposed for this aim, such as BioSeq-Analysis (47), Pse-in-One (48), and Pse-Analysis (49) (**Figure 2**).

## Author Contributions

ES and AA conceived of the presented idea. SAJ, AF, and SSH developed the theory and performed the computations. GP and AM verified the methods. SMH and SM provided the initial draft of the manuscript. All authors discussed the results and contributed to the final manuscript.

## Conflict of Interest

The authors declare that the research was conducted in the absence of any commercial or financial relationships that could be construed as a potential conflict of interest.

## Publisher’s Note

All claims expressed in this article are solely those of the authors and do not necessarily represent those of their affiliated organizations, or those of the publisher, the editors and the reviewers. Any product that may be evaluated in this article, or claim that may be made by its manufacturer, is not guaranteed or endorsed by the publisher.
